# Role of *IFNL4* Genotype in Hepatic Inflammation and Fibrosis due to Metabolic Dysfunction-Associated Steatotic Liver Disease

**DOI:** 10.21203/rs.3.rs-11009884/v1

**Published:** 2026-09-14

**Authors:** Callie J. Zaborenko, Aaron Hakim, Tae-Hwi Schwantes-An, Harish Gopalakrishna, David E. Kleiner, Marc G. Ghany, Ruth M. Pfeiffer, Chaowapong Jarasvaraparn, Tiebing Liang, Kung-Hung Lin, Ludmila Prokunina-Olsson, Eduardo Vilar-Gomez, Niharika R Samala, Naga Chalasani, Thomas O’Brien

**Affiliations:** Indiana University; Beth Israel Deaconess Medical Center; Indiana University; NIDDK; National Cancer Institute; NIDDK; National Cancer Institute; Indiana University; Indiana University; Kaohsiung Medical University; National Cancer Institute; Indiana University; Indiana University; Indiana University; National Cancer Institute

**Keywords:** IFNL4, interferon lambda, fatty liver disease, genetics

## Abstract

**Background and Aims:**

In patients infected with hepatitis C virus, genetic polymorphisms within or near the interferon lambda 4 gene (*IFNL4*) predict the risk of hepatic inflammation and fibrosis. Several studies have reported that these polymorphisms are also associated with inflammation and fibrosis in patients with fatty liver disease. To further explore this question, we examined whether the functional *IFNL4*-ΔG allele, which generates the IFN-λ4 protein, was associated with decreased severity of hepatic inflammation, fibrosis and related measures across three cohorts of participants with metabolic dysfunction-associated steatotic liver disease (MASLD).

**Approach and Results:**

Among 1786 non-Hispanic, white adults with biopsy-confirmed MASLD enrolled in the Nonalcoholic Steatohepatitis Clinical Research Network cohort (NASH CRN), the *IFNL4*-ΔG allele did not associate with histologic measures of disease severity: fibrosis - adjusted odds ratio [aOR] = 0.98, 95% confidence interval [CI] 0.86–1.11, p = 0.74; steatosis - aOR = 0.95, 95% CI 0.83–1.08, p = 0.40; lobular inflammation - aOR = 1.03, 95% CI 0.90–1.19, p = 0.63; NAFLD Activity Score - aOR = 1.00, 95% 0.88–1.13, p = 0.98. Results were similar in 358 Hispanic adults and 897 children enrolled in NASH CRN. In the Indiana University Nonalcoholic Fatty Liver Disease cohort (n = 606), *IFNL4*-ΔG was not associated with liver stiffness measurement (LSM). In the HIV NASH CRN cohort (n = 645), the *IFNL4*-ΔG allele associated with increased LSM in non-Hispanic, white, but not in non-Hispanic, black individuals.

**Conclusions:**

In contrast to some previous studies, this investigation of > 4,000 individuals provides no evidence that the *IFNL4*-ΔG allele is associated with decreased hepatic inflammation or fibrosis in patients with MASLD.

## INTRODUCTION

Metabolic dysfunction-associated steatotic liver disease (MASLD) has become the most common chronic liver disease worldwide.^1^ The severity of hepatic fibrosis in MASLD is a key factor that determines clinical outcomes, including progression to end-stage liver disease and hepatocellular carcinoma.^2^ The inflammatory response to a variety of pathogens or injurious agents is a major driver of liver fibrogenesis.^3^ Interferons play an important role in host defense and the inflammatory response through the induction of interferon-stimulated genes.^4^

In 2013, the discovery of the interferon lambda 4 gene (*IFNL4)* was reported.^5^ The production of the IFN-λ4 protein is controlled by a heritable dinucleotide frameshift variant, denoted as *IFNL4*-ΔG/TT (rs368234815). The *IFNL4*-ΔG allele generates the protein, while the TT allele abrogates production of IFN-λ4.^5^ Multiple variants in the interferon lambda genetic locus at chromosomal location 19q13.2 are in strong linkage disequilibrium (LD) with *IFNL4*-ΔG/TT, including the *IFNL4* rs12979860 variant, a single nucleotide polymorphism (SNP) marker located in the first intron of *IFNL4* that is associated with hepatitis C virus (HCV) clearance.^6–9^ In several studies, genotype for the *IFNL4*-ΔG/TT variant predicted spontaneous clearance of HCV and sustained virologic response after treatment for HCV better than genotype for *IFNL4* rs12979860.^5,10,11^ Individuals carrying the functional *IFNL4*-ΔG allele had poorer rates of spontaneous and treatment-related HCV clearance compared to those who were homozygous for the non-functional *IFNL4*-TT allele.

Studies in HCV-infected patients also demonstrated that the same genetic polymorphisms within *IFNL4* that predict HCV clearance are associated with hepatic inflammation and fibrosis in patients with chronic hepatitis C, however, for those outcomes, the *IFNL4*-ΔG allele (or the linked *IFNL4* rs12979860 allele) was associated with decreased liver inflammation and fibrosis.^12–14^ *IFNL4* genetic variants have also been associated with hepatic inflammation and fibrosis in several,^12,15–17^ but not all,^18^ studies among individuals with MASLD.

To better define the relationship between *IFNL4* genetic variation and hepatic inflammation and fibrosis in individuals with MASLD, we studied over 4,000 patients enrolled in three independent cohorts, including adults with human immunodeficiency virus (HIV) infection and children.

## METHODS

### Description of Cohorts and Participants:

A total of 4,309 patients with MASLD were recruited from three cohorts: 1) the Nonalcoholic Steatohepatitis Clinical Research Network (NASH CRN), a large, multicenter, prospective study of patients enrolled based on clinical criteria and liver biopsy; 2) the Human Immunodeficiency Virus Nonalcoholic Steatohepatitis Clinical Research Network (HIV NASH CRN), a prospective cohort of individuals with documented HIV; and 3) the Indiana University Nonalcoholic Fatty Liver Disease (IU-NAFLD) study, a cohort of non-invasively assessed MASLD or Metabolic Dysfunction-Associated Steatohepatitis (MASH) at a tertiary medical center. Self-reported ancestry, age in years, sex, and body mass index (BMI) were collected on all respondents at the baseline clinic visit in each cohort. All research was conducted in accordance with both the Declarations of Helsinki and Istanbul, and was approved by the appropriate ethics or institutional review committees. Consent was given in writing by all subjects.

As previously described, NASH CRN is a multicenter, prospective cohort study of adult and pediatric patients with a diagnosis of MASLD based on clinical criteria and liver biopsy.^2,19^ In short, from 2009 to 2019, patients at least two years of age with a histologic diagnosis of MASLD or cryptogenic cirrhosis were enrolled across multiple centers in the United States. Patients with any of the following were excluded: daily consumption of more than 20 g of alcohol for women or 30 g for men, liver disease other than MASLD, prior liver transplant, or hepatocellular cancer. Overall, 2,144 adults and 897 children from NASH CRN were included in this analysis.

The HIV NASH CRN^20^ included adult people with HIV (PWH) who were prospectively enrolled at HIV clinics across the United States from 2021 to 2025. Inclusion criteria were age ≥ 18 years and HIV-1 RNA < 200 copies/mL. Those receiving antiretroviral therapy (ART) at the time of study entry were required to be on a stable regimen for a minimum of three months prior to enrollment.^21^ Participants were excluded if they had evidence of active hepatitis B, hepatitis C infection, or other liver diseases. Participants underwent clinical evaluation by a physician and vibration-controlled transient elastography (VCTE) for controlled attenuation parameters (CAP) and liver stiffness measurement (LSM). Respondents then completed the Alcohol Use Disorders Identification Test (AUDIT). Participants with fatty liver CAP ≥ 263 dB/m, AUDIT score < 8 (threshold for hazardous drinking), and at least one cardiometabolic factor were considered to have MASLD. In total, 645 PWH were included in this analysis. Liver biopsy was not part of the protocol for this study.

The IU-NAFLD cohort includes adults cared for at a tertiary medical center who have MASLD or MASH, based on abnormal liver echotexture on abdominal ultrasound or elevated liver enzymes in the absence of other identifiable causes of liver disease based on non-invasive lab tests.^22^ Liver biopsy was not required for study entry. Potential participants were excluded if they did not fast for at least three hours before transient elastography, had daily consumption of more than 20 g of alcohol for women or 30 g for men for three or more months in the past year, or had active substance abuse. Study enrollment began in 2024 and is still ongoing. This cohort enrolled 606 patients.

### Institutional Review and Informed Consent

Participants included in this paper have participated in the protocols conducted at Indiana University (PI: Naga Chalasani), through multicentered studies of the NASH CRN (PI: Naga Chalasani) and through multicentered studies of the HIV NASH CRN (PI: Naga Chalasani). Studies done at Indiana University were approved by the Institutional Review Board (IRB) of Indiana University – Indianapolis. The studies were also approved by an IRB at each participating site. Informed consent was obtained from all subjects and/or their legal guardian(s).

NASH CRN Site IRBs:

Ann & Robert H. Lurie Children’s Hospital of Chicago Institutional Review Board

Baylor College of Medicine Institutional Review Board

Benaroya Research Institute at Virginia Mason Institutional Review Board

Cincinnati Children’s Hospital Medical Center Institutional Review Board

Cleveland Clinic Foundation Institutional Review Board and Human Research Protections

Columbia University Institutional Review Board

Community Medical Centers Institutional Review Board (UC Fresno)

Duke University Health System Institutional Review Board

Emory University Institutional Review Board

Indiana University Institutional Review Board

Johns Hopkins Bloomberg School of Public Health Institutional Review Board

Johns Hopkins Medicine Institutional Review Board

MetroHealth Institutional Review Board (Case Western Reserve University)

Seattle Children’s Hospital & Regional Medical Center Institutional Review Board

St. Louis University Institutional Review Board

Swedish Medical Center Institutional Review Board

University of California, San Diego Human Research Protections Program

University of California, San Francisco Human Research Protection Program

Virginia Commonwealth University Office of Research Subjects Protection

HIV NASH Site IRBs:

Duke University Health System Institutional Review Board

Indiana University Institutional Review Board

Harris Health Institutional Review Board (UT Health McGovern Medical School at Houston)

Johns Hopkins Bloomberg School of Public Health Institutional Review Board

Johns Hopkins Medicine Institutional Review Board

University of Alabama Birmingham Institutional Review Board

University of California, San Diego Human Research Protections Program

University of California, San Francisco Human Research Protection Program

Virginia Commonwealth University Office of Research Subjects Protection

### MASLD-MASH Grading and Severity

In the NASH CRN cohort, liver biopsies were reviewed in a blinded fashion by the NASH CRN Pathology Committee and scored according to the NASH CRN Scoring System^23^. Fibrosis stage was assessed from 0 (no fibrosis) to 4 (cirrhosis). Steatosis was graded from 0–3, ballooning from 0–2, and lobular inflammation from 0–3; the sum of these measures comprised the NAFLD Activity Score (NAS). In NASH CRN, we tested the association between genotype for the *IFNL4*-ΔG/TT variant and the following outcomes: fibrosis stage, dichotomized (stage ≥ 3 vs. <3); fibrosis stage as an ordinal variable; steatosis grade; lobular inflammation grade; NAS score. Among adult NASH CRN participants, we also examined alanine aminotransferase (ALT) as a continuous variable.

Liver biopsy data were not available in the IU-NAFLD and HIV NASH CRN cohorts, where patients’ fatty liver disease was assessed non-invasively. Therefore, in those cohorts, we examined several indirect measures of hepatic inflammation or fibrosis including ALT; CAP; and LSM. CAP was dichotomized CAP ≥ 285 dB/m vs. CAP < 285 dB/m^24,25^. CAP ≥ 285 is a proxy for steatosis grade 3. LSM was dichotomized in two ways; 1) LSM ≥ 8 kPa vs. <8 kPa^26^, and 2) LSM ≥ 12 kPa vs. < 12 kPa^26^. LSM ≥ 8 is a proxy for fibrosis being stage 2 or higher, while LSM ≥ 12 is a proxy for fibrosis of stage 3 or higher.

#### *IFNL4* Genotyping

DNA was extracted from baseline blood samples using Puregene Blood Kit (QIAGEN, Cat. No. 158026) at the Indiana University School of Medicine. The genotyping for rs368234815 (*IFNL4-*ΔG/TT) was performed using the previously described protocol developed in the Laboratory of Translational Genomics at the National Cancer Institute.^5^ Genotyping was performed at Indiana University using a custom-designed TaqMan assay, which included the TaqMan primer/probe mix, Genotyping Master Mix (Applied Biosystems, Cat. No. 4371355) and sample DNA. The PCR amplification and allelic discrimination were conducted using the ABI SDS7700 system. Quality control was performed on a randomly selected 6.6% of the study samples, with a concordance rate of 99% (Table S1).

Genotype for the *IFNL4* rs12979860 polymorphism was generated for NASH CRN by the Regeneron Genetics Center (RGC). As previously described,^27^ common variants of interest (including *IFNL4* rs12979860) were genotyped using a SNP array. Quality control measures included: 1) retaining only variants and samples with ≥ 95% call rates; 2) removing one participant from each pair of genetically related individuals; and 3) p > 10^15^ by test for Hardy-Weinberg Equilibrium (HWE).

### Statistical Analyses

First, for each subsample, we estimated proportions and means of all variables used in subsequent analyses, including genotype and allele frequencies for the *IFNL4*-ΔG/TT polymorphism. We assessed deviations from the genotype proportions expected under HWE using a chi-square test. Next, to examine associations between *IFNL4*-ΔG/TT and the severity of liver disease, we used data at study entry in linear and logistic regression models in the cohorts. For continuous measures such as ALT, we used linear regression models with the genotype of *IFNL4*-ΔG/TT additively coded for the number of ΔG alleles (0, 1 or 2). For dichotomized outcomes, we used logistic regression, again coding the variant additively. For multivariable analyses, we adjusted models for age and self-reported sex. To address confounding between *IFNL4*-ΔG/TT and ancestry or age group (i.e. adults vs. children), we stratified analyses based on self-reported ancestry (non-Hispanic White [NHW], non-Hispanic Black [NHB], and Hispanic) and age group. We performed a total of 37 tests in the primary analysis (Table 2). We present nominal p-values in the tables but when interpreting results, we used a Bonferroni adjustment for multiple testing, with a p-value < 0.0014 (i.e., 0.05/37) considered to be significant.

### Comparative analyses

As our results differed from those seen in some previous studies, we sought to replicate analyses from three such studies.^12,15,17^ We conducted these analyses among White adults enrolled in NASH CRN because that group most closely matched the ancestry of subjects in the original publications and because NASH CRN collected data on many of the same or similar covariates as in the original publications. Where possible, variables used in the replication analyses were coded to match those reported in the methods section of the original papers. Regarding *IFNL4* genotype, two studies examined genotype for *IFNL4* rs12979860 (coded as C/C vs. T/C or T/T) rather than *IFNL4*-ΔG/TT, therefore, our comparison for those studies was based on *IFNL4* rs12979860. Instances where variable coding or measurement differed between our analysis and the replication or where a variable was missing in our dataset are noted in table footnotes. Model selection followed that in the original papers. As a result, some of our replicative multivariate models include only variables that were significant in the original univariate analysis (regardless of whether that variable was significant in our univariate analysis), while other models include additional variables.

## RESULTS

### Participant Characteristics

Among the 4,309 participants across the three cohorts, five were removed due to missing data for *IFNL4*-ΔG/TT and 12 were removed due to missing data for a covariate, for a final total of 4,292 participants. A summary of the baseline characteristics of participants in each cohort and subgroups is shown in Table 1. The two NASH CRN adult subsamples (NHW and Hispanic adults) were majority female, while all other adult subsamples and both pediatric subsamples were predominantly male. Mean ages among adult subsamples were 46.1–54.4 years. Among children enrolled in NASH CRN, the mean age was 14.0 years for NHW and 12.5 years for Hispanics. The mean BMI across the subsamples ranged from 29.4 to 35.7.

Information from liver biopsy was available for NASH CRN adults and children. Across all NASH CRN subgroups, more than two-thirds of participants had a fibrosis score < 3. For lobular inflammation, across the NASH CRN subsamples 48% or more participants had a score of 0–1. Across the NASH CRN subsamples, the most common lobular inflammation score was 1. About a quarter of NASH CRN adults had a steatosis score of 3, compared to about half of pediatric NASH CRN participants. Over half of the participants in each subsample had NAS ≤ 4.

For the IU-NAFLD and HIV-NASH cohorts, hepatic fibrosis and inflammation were measured indirectly. Among IU-NAFLD participants, more than 70% had a CAP ≥ 285. More than half had an LSM ≥ 8 and more than one-third had an LSM ≥ 12. The mean ALT for this cohort was 58. For both HIV NASH CRN subgroups, values for CAP, LSM and ALT were lower than those for the IU-NAFLD participants.

The frequency of the functional *IFNL4*-ΔG allele ranged from 30.6%-32.8% in the NHW groups and was higher among the Hispanic participants in NASH CRN (adults, 39.1%; children, 45.8%). For the HIV NASH CRN NHB subgroup, the frequency of the *IFNL4*-ΔG allele was 63.6%, which is consistent with the higher reported frequency of this allele among individuals of African ancestry.^5^ The distribution of genotype proportions violated expectations under HWE only in the HIV NASH CRN NHB subgroup (p = 0.003), in which the proportion of heterozygotes was lower than expected given the frequency of the *IFNL4*-ΔG allele.

### Multivariable Analyses

To examine associations of *IFNL4*-ΔG/TT genotype with liver disease severity, we created multivariable models that were adjusted for age, sex, and BMI (Table 2). These adjusted results differed little from results based on univariable models (**Table S2**). Neither fibrosis ≥ 3 nor fibrosis treated as an ordinal variable was significantly associated with *IFNL4*-ΔG/TT genotype in any of the NASH CRN population subsets (e.g., NASH CRN NHW adults: fibrosis ordinal, adjusted odds ratio [aOR] = 0.98; 95% confidence interval [CI] 0.86–1.11; p = 0.74; fibrosis 3+, aOR = 1.04; 95% CI 0.89–1.21; p = 0.66). Similarly, there were no associations between *IFNL4*-ΔG/TT genotype and lobular inflammation among subsets of adults or children enrolled in NASH CRN (Table 2; e.g., NHW adults: aOR = 1.03; 95% CI 0.90–1.19; p = 0.63). The ΔG allele was associated with a nominally statistically significantly lower odds of steatosis in the Hispanic adults from NASH CRN (aOR = 0.73; 95% CI 0.55–0.98; p = 0.04), but not in NASH CRN NHW adults (aOR = 0.95; 95% CI 0.83–1.08; p = 0.40). Among children enrolled in NASH CRN, steatosis trended toward lower odds ratios for the *IFNL4*-ΔG allele in both the NHW and Hispanic subsets, but neither finding was statistically significant. There were no significant associations for NAS among subsets of adults or children enrolled in NASH CRN (e.g., NHW adults: aOR = 1.00; 95% 0.88–1.13; p = 0.98).

As liver biopsy results were not available for participants in IU-NAFLD or HIV NASH CRN, we assessed steatosis and fibrosis in those cohorts indirectly by measurements for CAP and LSM, respectively. There were no significant associations between *IFNL4*-ΔG/TT genotype and CAP in IU-NAFLD NHW adults (CAP ≥ 285: aOR = 1.08; 95% CI 0.77–1.51; p = 0.66) or in either subgroup of the HIV NASH CRN cohort. For LSM, in HIV NASH CRN NHW participants the *IFNL4*-ΔG allele was associated with higher values, with each ΔG allele increasing the odds of having an LSM ≥ 8 (aOR = 1.72; 95% CI 1.05–2.83; p = 0.03) or ≥ 12 (aOR = 4.98; 95% CI 2.04–13.39; p = 7.2×10^−4^). However, there were no significant associations between the *IFNL4*-ΔG allele and LSM in HIV NASH CRN NHB adults (LSM ≥ 12: aOR = 1.10; 95% CI 0.43–3.08; p = 0.85) or among IU-NAFLD NHW adults (Table 2).

### Non-Obese Participants

It has been suggested that the association of interferon lambda genetic variation with hepatic inflammation and fibrosis due to MASH may be restricted to individuals who are not obese.^18^ We therefore performed an analysis that was restricted to participants with a BMI < 30. In the 436 NASH CRN NHW adults who met that criterion (Table 3), there were no associations between genotype for *IFNL4*-ΔG/TT and fibrosis, lobular inflammation, steatosis or NAS. In non-obese NASH CRN Hispanic adults (n = 114), there were no genotype associations with fibrosis, lobular inflammation or NAS, but a nominally statistically significant protective association with steatosis (aOR = 0.59; 95% CI 0.36–0.96; p = 0.04) as was found among the NASH CRN Hispanic adult participants overall. In the pediatric NASH CRN participants, we found no associations with fibrosis, lobular inflammation, steatosis or NAS in either the NHW or Hispanic subgroups.

Among the 115 non-obese IU-NAFLD NHW participants, there were no associations with either CAP or LSM. In the HIV NASH CRN cohort, consistent with the overall results, the *IFNL4*-ΔG allele was associated with higher odds of an LSM of ≥ 12 in 150 NHW adults (aOR = 4.50; 95% CI 1.26–19.26; p = 0.03). There was a similar association among the 213 NHB adults, but that result was not statistically significant (aOR = 4.62; 95% CI 0.51–235.18; p = 0.29).

### Replication of Prior Publications

In a cohort of 160 European patients, Petta and colleagues reported a robust association between genotype for *IFNL4* rs12979860, which is in strong LD with *IFNL4*-ΔG/TT, and lobular inflammation (rs12979860 C/C vs. T/C or T/T: aOR = 0.22; 95% CI 0.10–0.47; p < 0.001).^15^ In contrast, among the 1,691 NHW participants in NASH CRN, we found no association (**Table S4**: aOR = 1.07; 95% CI 0.88–1.31; p = 0.5). In another study among individuals of European ancestry (n = 488), Eslam et al found a significant relationship between *IFNL4* rs12979860 genotype and fibrosis stage ≥ 2 (*IFNL4* rs12979860 C/C vs. T/C or T/T: aOR = 1.66; 95% CI 1.15–2.43; p = 0.006)^12^, however, we did not replicate this result in 1,786 NHW NASH CRN participants (**Table S5**: aOR = 0.83; 95% CI 0.68–1.03, p = 0.09). In a third study (n = 947), Petta et al found a significant relationship between *IFNL4*-ΔG/TT (rs368234815) genotype and fibrosis stage ≥ 3 in NHW adults (*IFNL4* rs368234815 ΔG/ΔG vs. ΔG/TT vs. TT/TT: aOR = 1.69; 95% CI 1.19–2.46; p = 0.004)^15^, while in 1,691 NHW participants in NASH CRN we saw no association (**Table S6**: aOR = 0.96; 95% CI 0.81–1.13; p = 0.6). Similar to their findings in the entire cohort, Petta et al found an association between *IFNL4*-ΔG/TT (rs368234815) genotype and fibrosis stage ≥ 3 individuals with BMI < 30, (aOR = 1.81; 95% CI 1.11–3.07; p = 0.02), however, there was no similar association in NASH CRN (**Table S6**: aOR = 0.78; 95% CI 0.53–1.14; p = 0.2).

## DISCUSSION

A number of previous studies evaluated the association of the *IFNL4*-ΔG/TT genetic polymorphism or its proxies with hepatic inflammation and fibrosis related to MASLD (**Table S7**). Some studies provided evidence that the functional *IFNL4*-ΔG allele is associated with less hepatic inflammation and fibrosis,^12,15,17^ but another study failed to replicate that association.^18^ To examine this question further, we conducted a large multi-cohort study of *IFNL4*-ΔG/TT genotype and disease severity in individuals with MASLD. NASH CRN is a multicenter study in which hepatic inflammation and fibrosis are assessed based on tissue specimens collected by liver biopsy. Among > 2100 adults enrolled in this cohort we found no associations between *IFNL4*-ΔG/TT genotype and either hepatic inflammation or hepatic fibrosis. Similarly, our analysis of ~ 900 pediatric participants in NASH CRN yielded no associations with those outcomes. In the two other cohorts (IU and HIV-NASH), we assessed liver steatosis and fibrosis based on indirect methods (CAP and LSM, respectively). Consistent with the findings for NASH CRN, we found no association between *IFNL4*-ΔG/TT genotype and either CAP or LSM in the IU-NAFLD cohort. In the HIV NASH CRN cohort, which consisted of adults infected with HIV, the functional protein generating *IFNL4*-ΔG allele was associated with higher levels of LSM in the NHW adults (but not in the larger NHB group). However, in the previous studies that reported an association between *IFNL4* genotype and hepatic fibrosis, the *IFNL4*-ΔG allele (or its surrogates) was associated with lower rather than higher fibrosis stage. Thus, our analysis does not replicate previously reported significant associations between *IFNL4* genotype and hepatic inflammation or fibrosis.

The reason for these differences is unclear. The NASH CRN cohort has a higher BMI (mean, 34.9) than that reported from previous studies and it has been suggested that the association between *IFNL4* genotype and liver disease severity may be more apparent in non-obese patients with MASLD.^18^ To assess that hypothesis, we conducted analyses restricted to participants with a BMI < 30. Consistent with our findings in the entire dataset, we found no associations between biopsy-based measures of hepatic inflammation or fibrosis in the non-obese stratum of the NASH CRN cohort. In the HIV NASH CRN cohort, we saw associations between the *IFNL4*-ΔG allele and higher levels of LSM in both obese and non-obese NHW participants. To more directly compare our data to previous papers that reported an association^12,15,17^, we performed analyses based on the same covariates and genotype models used in the original papers, but again found no evidence of an association between *IFNL4* genotype and either hepatic inflammation or fibrosis. Taken together, our results provide no evidence for those associations in individuals with MASLD.

It is important to consider whether limitations in our study may have impaired our ability to reproduce those prior results. Errors in genotype results can affect study validity and potentially cause a ‘false negative’ result. However, in the present study, *IFNL4*-ΔG/TT was genotyped by a well-established assay and the genotype distributions for most population groups were highly consistent with those expected under HWE. We also found no associations in two replication analyses based on results for the *IFNL4* rs12979860 variant, which were obtained using a different genotyping platform. Our analysis relied on self-identified ancestry, and therefore, could be affected by uncontrolled population stratification; however, a previous study in the NASH CRN cohort replicated previously observed associations between genotype for the *PNPLA3* rs738409 variant and hepatic inflammation and fibrosis.^28^

It is also important to consider the strengths of our study. The present study is considerably larger than any previous study of *IFNL4* genotype and hepatic inflammation or fibrosis due to MASLD, and, therefore, had sufficient statistical power to replicate previous positive findings. Misclassification of outcome variables could also reduce the ability to find an association. In the NASH CRN cohort, inflammation and fibrosis were measured directly from hepatic tissue, which is the gold standard for those measures, however, for IU-NAFLD and HIV NASH CRN, we used surrogate markers for steatosis and fibrosis, which is a limitation of the analyses in those cohorts. Given these strengths, it seems likely that our results reflect the lack of a association in these US populations.

To our knowledge, this is the first report to examine the relationship between genotype for the *IFNL4*-ΔG/TT polymorphism and measures of hepatic fibrosis in individuals with both HIV infection and MASLD. In the HIV NASH CRN cohort, the presence of the *IFNL4*-ΔG allele, which generates the IFN-λ4 protein, was associated with high LSM in NHW (but not NHB) participants. That observed relationship is opposite to previous reports that associated the functional *IFNL4*-ΔG allele (or surrogates) with decreased hepatic inflammation and fibrosis due to MASH or HCV,^12–15,17^ and inconsistent with our findings of no association between *IFNL4*-ΔG/TT and hepatic fibrosis in the NASH CRN cohort. It seems possible that the association observed in the HIV NASH CRN NHW subjects is due to chance. In conclusion, while several prior studies reported associations of *IFNL4*-ΔG with decreased hepatic inflammation and fibrosis in individuals with MASLD, our larger study failed to confirm those relationships. An explanation for these inconsistent results is not obvious, however, in contrast to other studies on this question, neither our study nor another study that enrolled participants in the United States^18^ found these associations. Additional studies may be needed to evaluate the apparently heterogenous relationship of *IFNL4* genotype with liver inflammation and fibrosis in patients with MASLD.

## Figures and Tables

**Table 1:** Characteristics of the Cohorts

Cohort	NASH CRN		IU-NAFLD	HIV NASH CRN		NASH CRN	
Population	Biopsy-proven MASLD (adults)		Adult MASLD characterized clinical, lab, and imaging	Adult PWH		Biopsy-proven MASLD (children)	
MASLD characterization	Liver histology		VCTE ± LSM	VCTE		Liver histology	
Subsample	NHW Adult (N = 1786)	H Adult (N = 358)	NHW Adult (N = 606)	NHW Adult (N = 240)	NHB Adult (N = 405)	NHW Pediatric (N = 219)	H Pediatric (N = 678)
Sex							
Male	36.8%	38.8%	62.7%	87.5%	60.5%	73.5%	72.9%
Female	63.2%	61.2%	37.3%	12.5%	39.5%	26.5%	27.1%
Age							
Mean (SD)	51.1 (11.6)	46.1 (12.8)	52.7 (13.3)	54.4 (10.8)	52.7 (12.1)	14.0 (2.45)	12.5 (2.77)
[Min, Max]	[17.3, 78.5]	[17.6, 73.6]	[18.0, 84.0]	[26.0, 81.0]	[21.4, 78.0]	[7.38,18.0]	[4.70,18.4]
BMI							
Mean (SD)	34.9 (6.56)	33.3 (5.84)	35.7 (7.04)	29.4 (6.58)	30.4 (6.97)	34.1 (6.61)	31.4 (6.08)
[Min, Max]	[15.8, 66.9]	[20.0, 54.3]	[17.3, 69.8]	[18.1, 58.8]	[17.7, 54.8]	[21.4, 67.7]	[16.7, 64.4]
Fibrosis (binary)							
<3	67.1%	76.8%				86.8%	85.8%
3+	32.5%	22.6%				12.8%	14.2%
Missing	0.4%	0.6%				0.5%	0%
Fibrosis (ordinal)							
0	23.1%	29.9%				29.7%	34.5%
1	24.5%	28.5%				41.1%	38.9%
2	19.4%	18.4%				16.0%	12.4%
3	21.4%	15.1%				11.4%	12.8%
4	11.1%	7.5%				1.4%	1.3%
Missing	0.4%	0.6%				0.5%	0%
Lobular Inf.							
0	1.0%	1.4%				0.5%	0.4%
1	54.9%	46.4%				63.5%	55.3%
2	34.2%	39.1%				32.9%	36.9%
3	10.0%	13.1%				3.2%	7.4%
Steatosis							
0	5.3%	7.0%				3.2%	2.9%
1	36.5%	41.3%				18.7%	27.6%
2	33.0%	27.4%				27.9%	29.2%
3	25.2%	24.3%				50.2%	40.3%
NAS							
0	0.7%	0.3%				0%	0.1%
1	2.4%	3.6%				3.2%	2.4%
2	13.6%	14.0%				10.0%	15.9%
3	15.6%	15.9%				17.4%	18.9%
4	23.0%	19.3%				30.6%	24.6%
5	18.6%	20.9%				19.6%	21.2%
6	14.3%	15.4%				13.2%	11.7%
7	9.5%	8.1%				4.6%	3.4%
8	2.4%	2.5%				1.4%	1.8%
CAP 285							
<285			19.0%	52.9%	71.6%		
>=285			72.8%	47.1%	28.4%		
Missing			8.3%	0%	0%		
LSM 8							
<8			40.6%	77.5%	91.4%		
>=8			51.2%	22.5%	8.6%		
Missing			8.3%	0%	0%		
LSM 12							
<12			58.6%	93.3%	97.5%		
>=12			33.2%	6.7%	2.5%		
Missing			8.3%	0%	0%		
ALT							
Mean (SD)	67.0 (49.0)	73.6 (57.4)	57.8 (45.9)	36.7 (28.1)	23.0 (16.4)	98.5 (72.2)	111 (92.4)
[Min, Max]	[10.0, 514]	[11.0, 460]	[6.00, 330]	[8.00, 193]	[5.00, 136]	[7.00, 714]	[16.0, 696]
Missing	0.8%	1.4%	14.9%	0.4%	1.2%	0%	0%
*IFNL4-*Δ*G/TT (rs368234815)*							
Frequency of ΔG	32.8%	39.1%	30.6%	32.5%	63.6%	32.4%	45.8%
Genotype Frequency							
TT/TT	44.9%	36.3%	48.3%	44.6%	16.8%	47.0%	29.9%
ΔG/TT	44.7%	49.2%	42.1%	45.8%	39.3%	41.1%	48.5%
ΔG/ΔG	10.4%	14.5%	9.6%	9.6%	44.0%	11.9%	21.5%
HWE p-value	0.59	0.58	0.85	0.56	**0.003**	0.36	0.64

ALT - Alanine Aminotransferase; BMI - Body Mass Index; CAP - Controlled Attenuation Parameter; H – Hispanic; HIV - Human Immunodeficiency Virus; HWE - Hardy-Weinberg Equilibrium; Inf. – Inflammation; IU - Indiana University; LSM - Liver Stiffness Measurement; NAS - NAFLD Activity Score MASLD - Metabolic dysfunction-Associated Steatotic Liver Disease; Max – Maximum; Min – Minimum; NAFLD - Non-Alcoholic Fatty Liver Disease; NAS - NAFLD Activity Score; NASH CRN- Non-Alcoholic Steatohepatitis Clinical Research Network; NHB - Non-Hispanic Black; NHW - Non-Hispanic White; PWH - People With HIV; SD - Standard Deviation; VCTE - Vibration Controlled Transient Elastography

**Table 2:** Multivariable models for associations between *IFNL4*-ΔG/TT genotype and severity of liver disease

	NASH CRN				IU-NAFLD		HIV NASH CRN				NASH CRN			
	NHW Adult		Hispanic Adult		NHW Adult		NHW Adult		NHB Adult		NHW Ped		Hispanic Ped	
	(N = 1786) ^a^		(N = 358) ^a^		(N = 606) ^a^		(N = 240) ^a^		(N = 405) ^a^		(N = 219) ^a^		(N = 678) ^a^	
	Odds Ratios (95% CI)	*p*	Odds Ratios (95% CI)	*p*	Odds Ratios (95% CI)	*p*	Odds Ratios (95% CI)	*p*	Odds Ratios (95% CI)	*p*	Odds Ratios (95% CI)	*p*	Odds Ratios (95% CI)	*p*
Dependent Variables														
Fibrosis	1.04	0.66	1.03	0.88							0.84	0.58	1.12	0.47
(< 3vs ≥ 3)	(0.89–1.21)		(0.70–1.52)								(0.44–1.53)		(0.82–1.53)	
Fibrosis	0.98	0.74	1.13	0.40							1.19	0.32	1.03	0.76
(Ordinal 0–4)	(0.86–1.11)		(0.85–1.48)								(0.84–1.69)		(0.85–1.25)	
Lobular	1.03	0.63	1.03	0.87							0.90	0.61	0.90	0.31
Inflammation	(0.90–1.19)		(0.76–1.38)								(0.59–1.35)		(0.73–1.10)	
Steatosis	0.95	0.40	0.73	**0.04**							0.76	0.14	0.84	0.08
	(0.83–1.08)		(0.55–0.98)								(0.52–1.10)		(0.69–1.02)	
NAS	1.00	0.98	0.91	0.48							0.93	0.68	0.86	0.11
	(0.88–1.13)		(0.69–1.19)								(0.66–1.32)		(0.71–1.03)	
CAP > = 285					1.08	0.66	1.51	0.08	1.33	0.10				
					(0.77–1.51)		(0.96–2.41)		(0.95–1.88)					
LSM > = 8					1.00	0.98	1.72	**0.03**	1.22	0.44				
					(0.76–1.32)		(1.05–2.83)		(0.74–2.07)					
LSM > = 12					1.08	0.57	4.98	**7.2E-4**	1.1	0.85				
					(0.82–1.43)		(2.04–13.39)		(0.43–3.08)					
	Coef. (95% CI)	*p*	Coef. (95% CI)	*p*	Coef. (95% CI)	*p*	Coef. (95% CI)	*p*	Coef. (95% CI)	*p*	Coef. (95% CI)	*p*	Coef. (95% CI)	*p*
ALT	1.62	0.35	4.12	0.35	1.58	0.60	0.90	0.75	2.06	0.06				
(Continuous)	(−1.77–5.02)		(−4.62–12.86)		(−4.39–7.55)		(−4.72–6.52)		(−0.05–4.17)					

*IFNL4*-ΔG/TT is coded based on an additive genetic model, as TT/TT = 0, ΔG/TT = 1, and ΔG/ΔG = 2. All models adjust for age, sex, and BMI.

a The sample size reported is the maximum potential sample size, the true sample size varies by outcome.

b This result passed the Bonferroni correction and has a Bonferroni adjusted p-value of 0.03

ALT - Alanine Aminotransferase; CAP - Controlled Attenuation Parameter; H – Hispanic; HIV - Human Immunodeficiency Virus; Inf. – Inflammation; IU - Indiana University; LSM - Liver Stiffness Measurement; NAS - NAFLD Activity Score MASLD - Metabolic dysfunction-Associated Steatotic Liver Disease; NAFLD - Non-Alcoholic Fatty Liver Disease; NAS - NAFLD Activity Score; NASH CRN- Non-Alcoholic Steatohepatitis Clinical Research Network; NHB - Non-Hispanic Black; NHW - Non-Hispanic White; PWH - People With HIV; SD - Standard Deviation; VCTE - Vibration Controlled Transient Elastography

**Table 3:** Multivariable models for associations between *IFNL4*-ΔG/TT genotype and severity of liver disease among participants with a body mass index < 30

	NASH CRN				IU-NAFLD		HIV NASH CRN				NASH CRN			
	NHW Adult		Hispanic Adult		NHW Adult		NHW Adult		NHB Adult		NHW Ped		Hispanic Ped	
	(N = 436) ^a^		(N = 114) ^a^		(N = 115) ^a^		(N = 150) ^a^		(N = 213) ^a^		(N = 114) ^a^		(N = 308) ^a^	
	Odds Ratios (95% CI)	*p*	Odds Ratios (95% CI)	*p*	Odds Ratios (95% CI)	*p*	Odds Ratios (95% CI)	*p*	Odds Ratios (95% CI)	*p*	Odds Ratios (95% CI)	*p*	Odds Ratios (95% CI)	*p*
Dependent Variables														
Fibrosis	1.24	0.22	0.91	0.79							1.39	0.58	1.12	0.61
(< 3, ≥ 3)	(0.88–1.75)		(0.44–1.82)								(0.43–4.45)		(0.72–1.77)	
Fibrosis	1.14	0.32	1.02	0.95							1.43	0.33	1.05	0.76
(Ordinal 0–4)	(0.88–1.48)		(0.63–1.64)								(0.70–2.98)		(0.78–1.42)	
Steatosis	1.20	0.19	0.59	**0.04**							0.62	0.24	0.85	0.29
	(0.92–1.56)		(0.36–0.96)								(0.28–1.35)		(0.64–1.14)	
NAS	1.16	0.26	0.79	0.32							1.29	0.48	0.88	0.35
	(0.90–1.50)		(0.49–1.25)								(0.64–2.64)		(0.66–1.16)	
Lobular	1.07	0.63	1.30	0.54							1.05	0.86	0.90	0.52
Inflammation	(0.80–1.43)		(0.56–3.07)								(0.63–1.74)		(0.65–1.24)	
CAP > = 285					1.14	0.64	1.63	0.09	0.83	0.54				
					(0.65–2.02)		(0.94–2.87)		(0.47–1.52)					
LSM > = 8					1.17	0.59	1.57	0.19	1.09	0.82				
					(0.65–2.12)		(0.80–3.09)		(0.53–2.39)					
LSM > = 12					1.02	0.96	4.50	**0.03**	4.62	0.29				
					(0.51–1.98)		(1.26–19.26)		(0.51–235.18)					
	Coef. (95% CI)	*p*	Coef. (95% CI)	*p*	Coef. (95% CI)	*p*	Coef. (95% CI)	*p*	Coef. (95% CI)	*p*	Coef. (95% CI)	*p*	Coef. (95% CI)	*p*
ALT	3.16	0.43	1.82	0.81	3.42	0.59	−0.78	0.79	2.22	0.08				
(Continuous)	(−4.76–11.09)		(−13.10–16.75)		(−9.06–15.91)		(−6.56–5.01)		(−0.26–4.70)					

*IFNL4*-ΔG/TT is coded based on an additive genetic model, as TT/TT = 0, ΔG/TT = 1, and ΔG/ΔG = 2. All models adjust for age and sex.

a The sample size reported is the maximum potential sample size, the true sample size varies by outcome.

ALT - Alanine Aminotransferase; CAP - Controlled Attenuation Parameter; H – Hispanic; HIV - Human Immunodeficiency Virus; Inf. – Inflammation; IU - Indiana University; LSM - Liver Stiffness Measurement; NAS - NAFLD Activity Score MASLD - Metabolic dysfunctionAssociated Steatotic Liver Disease; NAFLD - Non-Alcoholic Fatty Liver Disease; NAS - NAFLD Activity Score; NASH CRN- Non-Alcoholic Steatohepatitis Clinical Research Network; NHB - Non-Hispanic Black; NHW - Non-Hispanic White; PWH - People With HIV; SD - Standard Deviation; VCTE - Vibration Controlled Transient Elastography

## Data Availability

Requests for data sharing will be considered on a request-by-request basis, in compliance with participant consent and regulatory requirements. Data sharing requests should be addressed to Dr. Naga Chalasani at: nchalasa@iu.edu
